# Cooperation and Lateral Forces: Moving Beyond Bottom-Up and Top-Down Drivers of Animal Population Dynamics

**DOI:** 10.3389/fpsyg.2022.768773

**Published:** 2022-02-02

**Authors:** Ying-Yu Chen, Dustin R. Rubenstein, Sheng-Feng Shen

**Affiliations:** ^1^Biodiversity Research Center, Academia Sinica, Taipei, Taiwan; ^2^Department of Ecology, Evolution and Environmental Biology, Center for Integrative Animal Behavior, Columbia University, New York, NY, United States; ^3^Institute of Ecology and Evolutionary Biology, College of Life Science, National Taiwan University, Taipei, Taiwan

**Keywords:** intraspecific cooperation, social evolution, environmental quality, population dynamics, resilience

## Abstract

Biologists have long known that animal population dynamics are regulated by a combination of bottom-up (resource availability) and top-down forces (predation). Yet, economists have argued that human population dynamics can also be influenced by intraspecific cooperation. Despite awareness of the role of interspecific cooperation (mutualism) in influencing resource availability and animal population dynamics, the role of intraspecific cooperation (sociality) under different environmental conditions has rarely been considered. Here we examine the role of what we call “lateral forces” that act within populations and interact with external top-down and bottom-up forces in influencing population dynamics using an individual-based model linking environmental quality, intraspecific cooperation, and population size. We find that the proportion of cooperators is higher when the environment is poor and population sizes are greatest under intermediate resources levels due to the contrasting effects of resource availability on behavior and population size. We also show that social populations are more resilient to environmental change than non-social ones because the benefits of intraspecific cooperation can outweigh the effects of constrained resource availability. Our study elucidates the complex relationship between environmental harshness, cooperation, and population dynamics, which is important for understanding the ecological consequences of cooperation.

## Introduction

The abundance or carrying capacity of animal populations is often determined by top-down forces like predation pressure or bottom-up forces like resource availability, both of which can be influenced by environmental conditions (Schluter and Repasky, 1991; Anne and Rudy, 1997; Walankiewicz, 2002; Hopfenberg, 2003; Berryman, 2004; Rutz and Bijlsma, 2006; Melis et al., 2009). The concept of bottom-up forces extends the view of resource-constrained populations proposed by the economist Thomas Malthus over two centuries ago (Malthus, 1798). Not only is Malthus’ view on resource-constrained population dynamics still widely held in ecology (Lomnicki, 1988; May and McLean, 2007; Gotelli, 2008; Molles, 2016), his view on the human struggle for existence remains central to the theory of evolution by natural selection (Darwin, 1859). After the industrial revolution, however, the growth of the world’s population prompted economists to reconsider the role of resources in human population dynamics (Brown, 1954; Cépède et al., 1964; Cohen, 1995). More than a half century ago, the economist Boserup (1965) further proposed that high population density stimulated human cooperation in order to improve agricultural efficiency, thereby increasing resource supply to match the needs of a growing population. In contrast to the views of Malthus, Boserup hypothesized that human populations can overcome resource constraints and thrive through cooperation. Whether human populations can actually escape from resource limitation through intraspecific cooperation remains a topic of great debate to this day (Lipton, 1989; Richerson and Boyd, 1997; Decker and Reuveny, 2005; Urdal, 2005; Demont et al., 2007; Willy et al., 2019; Egger et al., 2020).

As an extension of Boserup’s ideas, intraspecific cooperation can be considered to be a “lateral force” that acts within populations and interacts with external top-down and bottom-up forces to regulate population size. Although ecologists have long considered the role of *interspecific* cooperation (i.e., mutualism) in affecting resource availability and ultimately population dynamics (Bronstein, 1994; Stachowicz, 2001; Hay et al., 2004), the role of within-species social interactions in different environmental conditions has rarely been considered in studies of population dynamics of living organisms other than humans, where the role of *intraspecific* cooperation has been widely discussed (Hamilton et al., 2009; Ellis et al., 2018). One exception comes from studies of microbes (Gore et al., 2009; de Vargas Roditi et al., 2013; Sanchez and Gore, 2013) that have explored the impact of intraspecific cooperation on population growth (Rainey and Rainey, 2003; Gore et al., 2009) or the interaction between intraspecific cooperation and population dynamics (Sanchez and Gore, 2013). Harsh environments are thought to favor intraspecific cooperation in microbes (Yurtsev et al., 2013; Bottery et al., 2016; Frost et al., 2018), as they do in other social animals (Rubenstein and Lovette, 2007; Jetz and Rubenstein, 2011; Lukas and Clutton-Brock, 2017; Firman et al., 2020), but how environment-associated intraspecific cooperation within populations or groups—what is often termed sociality—affects population dynamics remains largely unstudied in any organism.

Despite a large body of theoretical research examining the evolution of intraspecific cooperative behavior, the vast majority of models focus on how different cooperative strategies (e.g., tit-for-tat) affect the frequency of cooperators and free riders in populations by assuming that population size and environmental conditions are fixed (Axelrod and Hamilton, 1981; Ohtsuki et al., 2006; Traulsen and Nowak, 2006). Other studies have examined the effect of environmental conditions on the evolution of cooperation with population size held constant (Weitz et al., 2016; Pereda et al., 2017; Tilman et al., 2020), finding that environmental harshness tends to reduce resource availability and increase mortality, often favoring intraspecific cooperation (Andras et al., 2007; Requejo and Camacho, 2011). Recently, a small but growing number of studies have begun to dismiss the assumption of fixed population size (though they still assume that environmental conditions are fixed), finding primarily that cooperators are favored when population size is small but that free-riders are favored as the number of cooperators and the population size increases (Hauert et al., 2006). Finally, one series of studies considered both environmental conditions and population size change simultaneously, showing that while the presence of free-riders in harsh environments facilitates the evolution of cooperation, the presence of free-riders in environments abundant in resources actually helps to improve the use of resources by cooperators and results in free-riders essentially increasing the fitness of the entire population (MacLean et al., 2010; Lindsay et al., 2016). Thus, eco-evolutionary feedbacks between intraspecific cooperative behavior and population dynamics often induce the coexistence of cooperators and defectors (Hauert et al., 2006; Sanchez and Gore, 2013). Ultimately, clarifying the interactions between resource availability (a bottom-up force) and intraspecific cooperation (a lateral force) on population dynamics remains a challenge for theoretical biologists. Considering the relationships among population dynamics, intraspecific cooperation, and resource availability simultaneously is necessary to more fully understand how social species and populations respond to resource constraints and other environmental challenges.

**FIGURE 1:**
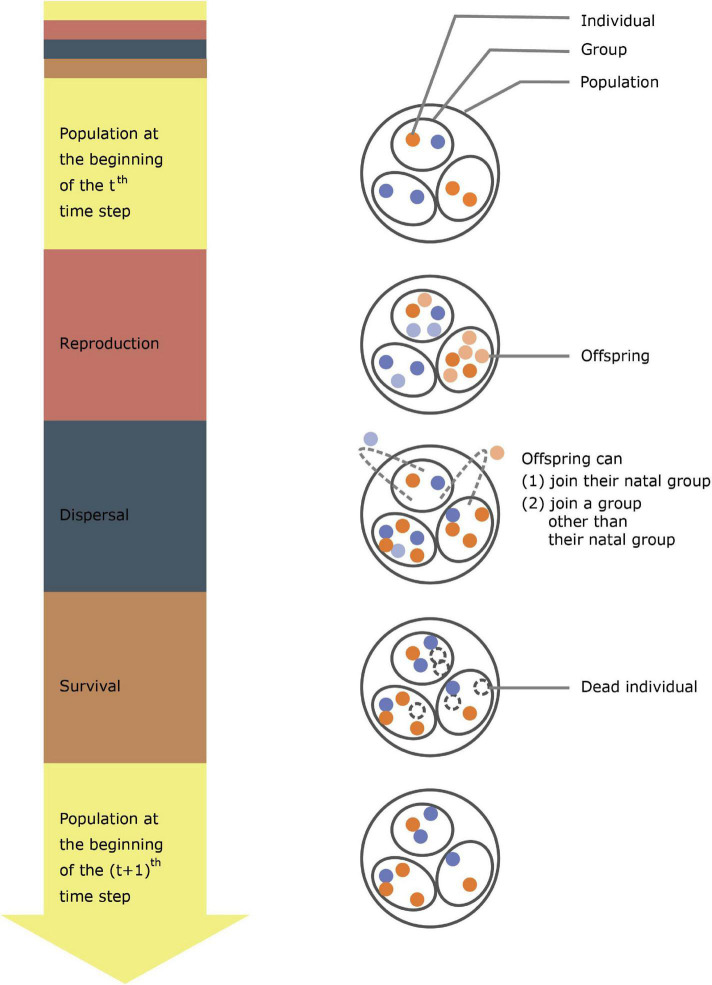
A schematic diagram for the model. The diagram shows the process that a population undergoes within one simulation time step. The red, blue, and brown portions in the arrow on the left represent reproduction, dispersal, and survival stages in an individual lifecycle, respectively. Orange and blue dots represent cooperators and non-cooperators, respectively, lighter colors represent newborn individuals, and dashed circles represent dead individuals.

Here, we develop a model with four main features. First, population size is not externally assumed, but instead emerges from the dynamics of birth and death processes that are influenced by both environmental conditions and individual behavioral strategies (Figure 1). Second, we define a social species as one in which individuals can exhibit cooperative behavior. We hypothesize that cooperative behavior is a continuous trait that is subject to natural selection. A high degree of cooperation represents a trait that invests more in generating benefits shared by all group members, including free-riders, at the cost of lowering individual fitness. We define individuals who invest in generating group benefits as cooperators and those that do not invest as free riders. Third, we allow for changes in the mean and variance of the environmental conditions so that our model not only accounts for variation in both population size and the environment, but also allows us to study the resilience of populations under different environmental conditions. Finally, we assume that the abundance of resources is mainly influenced by environmental conditions. However, cooperation can increase the group benefits in terms of value or efficiency of resource use (e.g., for cooperatively breeding species, the abundance of food is mainly affected by the environmental conditions in that year, and cooperation can improve the efficiency of foraging or catching prey at the same prey abundance). Thus, we expect that our model will complement long-standing interest by ecologists in the role of interspecific cooperation (and competition) over resources in shaping population dynamics in environments of varying quality (Bronstein, 1994; Stachowicz, 2001; Hay et al., 2004; Suweis et al., 2013).

## The Model

### General Description

We use an individual-based model to simulate the dynamics of structured populations (with non-fixed size) consisting of cooperators with varying degrees of cooperation and non-cooperators (free riders). Cooperators produce benefits (group resources that are shared equally by the group members) at a cost to themselves. Individuals have different genetically determined levels of cooperative investment, which determine the group resources that they generate. Group resources are essential for individuals’ reproduction. In other words, more cooperators generate greater group resources, which leads to a higher average reproductive rate of the group members. In contrast, non-cooperators provide no benefit to their groups and bear no cost; they simply consume the group resources.

For a comparison with social populations, we also model non-social populations consisting exclusively of non-cooperators. For simplicity, we consider asexual populations with a mutation rate (*m*) equal to 0.001. At the beginning of each simulation, population size (*N*) is set to 300, and all cooperators and free-riders are randomly divided into 90 groups. We assume that the interaction among individuals happens within groups. For completeness, we also model the scenario of non-structured populations and find that cooperation cannot evolve in such a scenario (Supplementary Figure 1), as has been found in other studies (Zhang and Hui, 2011).

Within every simulation time step, cooperators generate group resources, all individuals consume resources and reproduce, and some of them die. After individuals produce offspring, the offspring disperse and randomly join a group. The total number of time steps in a simulation is 10,000, which means that the evolutionary process lasts 10,000 years (roughly several thousand generations), to ensure that the system settles into relatively stable dynamics (Supplementary Figure 2), as suggested in the literature on non-linear dynamics (Strogatz, 2001). We record the proportion of cooperators in populations, the average degree of cooperation that an individual exhibits, the population size, and the total and per capita reproductive output of each group throughout the process. These properties spontaneously emerge from individual-level interactions.

All variables and parameters are summarized in Supplementary Table 1.

### Individual Life Cycles

The individuals will undergo the following process during each time step:

At the beginning, individuals equally share the group resources (*R*_*i, t*_, where *i* denotes the *i^th^* group and *t* denotes the *t^th^* time step). Therefore, when there are *N*_*i, t*_ individuals in the *i^th^* group, each individual’s resource consumption (*s*_*i, t*_) is equal to *R*_*i*,*t*_/*N*_*i*,*t*_. Group resources are determined by environmental resource availability (*R*_0_) and cooperative benefits (bK⁢∑j∅Ki,j). *b*_*K*_ denotes cooperation efficiency, and ∅Ki,j with eleven levels (0.0, 0.1, 0.2, ……, 1.0) denotes the *j^th^* individual’s degree of cooperation in the *i^th^* group. Group resources are a saturating function of cooperative benefits (bK⁢∑j∅Ki,j), which is analogous to the Monod equation (Monod, 1949):


(1) Ri,t=R0⁢(1+I⋅bK⁢∑j∅Ki,jI⁢R02+bK⁢∑j∅Ki,j),

where *I* is the maximum resource increment rate. This equation means that the more the group members invest in cooperation, the more resources they gain, but there is an upper limit for this beneficial effect. For the groups without cooperators generating benefits, *R*_*i*, *t*_ = *R*_0_.

Next, individuals produce offspring that disperse and randomly join a group. This occurs because in order to simplify the model, we do not assume an explicit spatial structure of the population. However, if offspring are more likely to join their natal patch, this will generally increase the frequency of cooperators within the population (Brauchli et al., 1999; Nadell et al., 2010). The number of offspring the *j^th^* individual can produce (reproductive rate *F*_*i, j, t*_) is a saturating function of the amount of resources it consumed in the form of the Monod equation (Monod, 1949), which also depends on the cost of cooperartion. In other words, an individual’s resource consumption positively influences its reproductive rate with an upper limit α, and if it is a cooperator, it will invest in cooperation at the cost of its own reproduction:


(2) Fi,j,t=α⁢(1-β⁢∅Ki,j)⋅si,t-MKs+(si,t-M),

where α denotes the maximum reproductive rate of an individual, β(0 < β ≤ 1) is defined as the percentage decrease in the reproductive rate caused by per unit cooperation degree (∅Ki,j), *M* is a constant and represents the metabolic consumption of an individual, and *K*_*s*_ is the “half-saturation constant,” which is the value of the individual energy for reproduction (*s*_*i*, *t*_ − *M*) at which the reproductive rate (*F*_*i, j, t*_) is half of its maximum.

Finally, the system determines whether individuals survive. The survival rate (*r*_*i, j, t*_) of the *j^th^* individual in the *i^th^* group decreases as it gets older:


(3) $$r_{i,j,t}=\text{c}\cdot e\,x\,p\,\left(-\frac{a\,g\,e_{i,j,t}}{a\,g\,e_{s\,t\,a\,n\,d\,a\,r\,d}}\right),$$

where c is a constant between 0 and 1. For the offspring born at the *t^th^* time step, *r*_*i*, *j*, *t*_ = *c* because *age*_*i*, *j*, *t*_ = 0.

### Populations in Fluctuating Environments

Next, we introduce environmental fluctuation into the system. Environmental resource availability (R0t) fluctuates periodically, which is described by a sine function:


(4) R0t=R00+A⁢s⁢i⁢n ⁢(2⁢π⁢tP),

where *A* denotes the amplitude and *P* denotes the period. We record the time series of the proportion of cooperators, the average degree of cooperation, and the population size. To derive the trend in population dynamics, we average the time series from 500 replicate simulations. We further use time-lagged cross-correlation (TLCC) to quantify synchrony between environmental fluctuation and population dynamics at the relatively stable state, and evaluate the variation of the population size by converting the time series of the population size variation standardized by the mean to the frequency spectra using a fast Fourier transformation (Dillon et al., 2016).

## Results

### Environmental Quality and the Evolution of Cooperation

**FIGURE 2:**
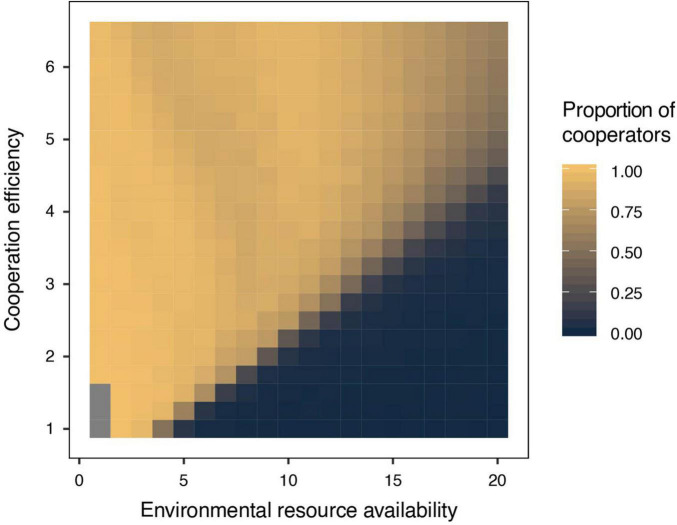
The proportion of cooperators in populations in relation to environmental resource availability and cooperation efficiency. Intraspecific cooperative behavior is more likely to evolve when environmental conditions are harsh (i.e., low environmental resource availability) and when cooperation efficiency, an intrinsic property of individuals, is high.

**FIGURE 3:**
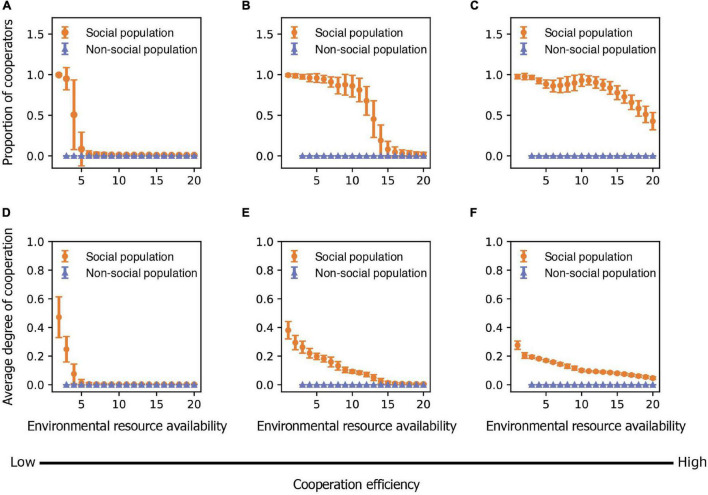
The evolution of cooperation and sociality is influenced by both variation in environmental resource availability and cooperation efficiency. **(A–C)** The mean proportion of cooperators in populations in relation to environmental resource availability as cooperation efficiency is **(A)** low (*b*_*K*_ = 1), **(B)** medium (*b*_*K*_ = 3), and **(C)** high (*b*_*K*_ = 5). **(D–F)** The mean degree of cooperation that individuals exhibit in relation to environmental resource availability as cooperation efficiency is **(D)** low (*b*_*K*_ = 1), **(E)** medium (*b*_*K*_ = 3), and **(F)** high (*b*_*K*_ = 5). Points represent means and bars represent standard deviations. Each mean and standard deviation is calculated on the output data of 500 simulations. The proportion of cooperators and degree of cooperation of non-cooperators are zero.

**FIGURE 4:**
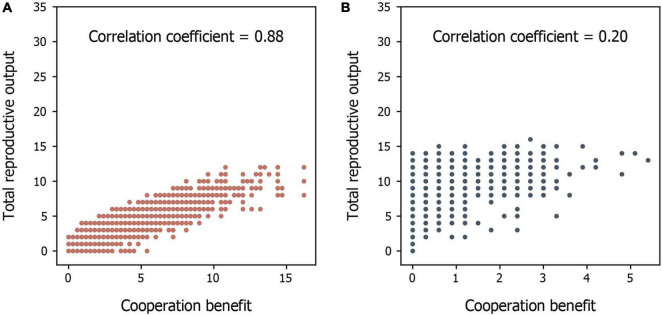
A comparison of the relationship between total reproductive output and cooperative benefits of groups in different environments. **(A,B)** The total reproductive output of groups in relation to their cooperative benefit in **(A)** harsh and **(B)** benign environments. Each point represents a group’s condition. The results are extracted from the early stage (the end of the 100th time step) of the simulations to see how cooperative benefits influence the reproductive output at the evolving stage.

We found that the evolution of cooperative behavior is determined jointly by the amount of available environmental resources and the benefits of intraspecific cooperation. Intraspecific cooperation, in terms of both the proportion of cooperators in the population and the average degree of cooperation that each individual performs, is more likely to evolve when environmental conditions are harsh (i.e., low resource availability) and when the benefits of cooperation are large (Figures 2, 3). This occurs because individuals are generally unable to produce offspring without the resources generated by cooperators in harsh environments (Figure 4A). Thus, free riders cannot persist without cooperators under harsh environmental conditions. In addition, although both cooperators and free riders share the group resources generated by cooperators (but only the cooperators have to pay personal costs), cooperators in groups with more group resources can still have more offspring than individuals in groups with fewer cooperators and group resources in harsh environments (Figure 4A). However, resources generated by cooperators play a smaller role in impacting reproduction in benign environments because available environmental resources are already abundant (Figure 4B). As a consequence, cooperation is maintained in harsh environments, particularly when the benefit of cooperating is high, a result consistent with previous models of environmental harshness and intraspecific cooperation (Epstein, 1998; Wang and Goldenfeld, 2011; Zhang and Hui, 2011; Smaldino et al., 2013).

### Joint Influence of Environmental Quality and Intraspecific Cooperation on Population Dynamics

**FIGURE 5:**
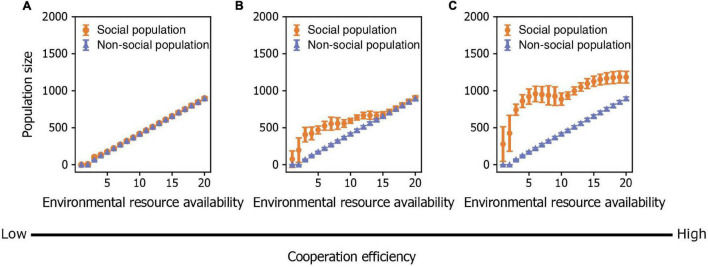
The combined effects of environmental resource availability and the degree of cooperation on population size. **(A–C)** Population size in relation to environmental resource availability when cooperation efficiency is **(A)** low (*b*_*K*_=1), **(B)** medium (*b*_*K*_=3), and **(C)** high (*b*_*K*_=5). Points represent means and bars represent standard deviations. Each mean and standard deviation is calculated on the output data of 500 simulations.

By exploring how environmental quality and intraspecific cooperation jointly influence population dynamics, we found that population size in social organisms is affected by environmental quality both directly in terms of resource availability and indirectly by its effect on the number of cooperators and the degree of intraspecific cooperation within the population. When the benefit of cooperating is small, population size is largely determined by environmental quality, resulting in a population that is similar in size to one without cooperators (Figure 5A). However, as the benefit of cooperating becomes greater, population size is determined by both environmental quality and intraspecific cooperation (Figure 5B). When the benefit of cooperating becomes very large, population size increases abruptly with an increase in environmental quality (i.e., an increase in resources) and then stays relatively constant (Figure 5C). This result can be explained by the fact that the average degree of intraspecific cooperation is also modulated by environmental quality in such a way that individuals are less cooperative in benign than in harsh environments. Therefore, the positive effect of additional resources in benign environments is canceled out by the negative effect of additional free riders. Moreover, additional cooperators in harsh environments compensate for any negative effects of resource scarcity. Furthermore, we showed that social populations possess an advantage in harsh environments with low resource availability because the benefit of cooperating is great enough to outweigh any effects of resource limitation on population size. This result implies that social populations can have wider ecological niches (i.e., can occur in environments with a wider range of resource availability) that non-social populations due solely to the fact that individuals cooperate.

### Stability of Population Dynamics in a Fluctuating Environment

**FIGURE 6:**
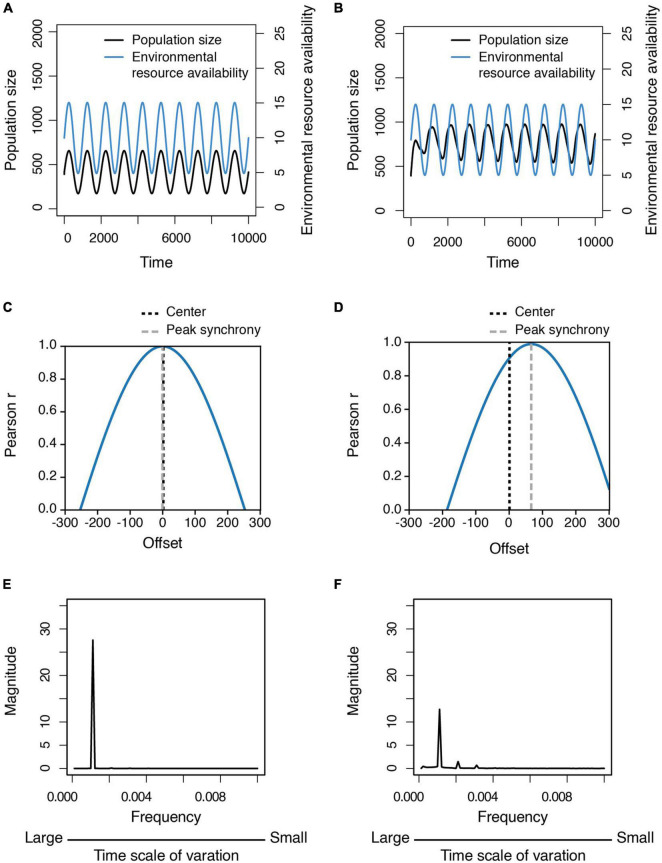
Population dynamics and relative variability in population size in a fluctuating environment. **(A,B)** Time series of the population size of **(A)** non-social populations and **(B)** social populations in the fluctuating environment. **(C,D)** The time-lagged cross correlation between the population dynamics and the environmental fluctuation for **(C)** non-social populations and **(D)** social populations. **(E,F)** The frequency spectra of the population size variation standardized by mean, which are derived from fast Fourier transformation, are shown for **(E)** non-social populations and **(F)** social populations. Each line is the average of the output data of 500 simulations.

Finally, we compared the population dynamics of social (i.e., cooperative) and non-social (i.e., non-cooperative) populations in a fluctuating environment, finding that the dynamics of non-social populations tend to synchronize with environmental fluctuation (Figures 6A,C), whereas the dynamics of social populations do not (Figures 6B,D). Population size increases after environmental conditions become extremely harsh, but decreases as conditions become more benign. In addition, the peak values in the frequency spectra of population size variation in non-social populations are higher than in social populations (Figures 6E,F), indicating that social populations are more stable than non-social ones in a fluctuating environment because the pattern of intraspecific cooperation is also modulated by environmental conditions (i.e., they are more cooperative in harsher environments), which can buffer the effect of changing environmental conditions on population size (Supplementary Figure 3).

## Discussion

Biologists have long known that environmental conditions influence the degree of both intraspecific (Jetz and Rubenstein, 2011; Shen et al., 2012, 2017; Sun et al., 2014) and interspecific cooperation (Maestre et al., 2009; He et al., 2013). Here, we show that environmental conditions also have an impact on the size and carrying capacity of social species in complex ways by affecting both the degree of intraspecific cooperation and the dynamics between cooperators and non-cooperators (i.e., free riders). Our model therefore provides a theoretical framework for understanding the ecological causes (e.g., environmental quality) and consequences (e.g., niche width) of intraspecific cooperation, extending previous work on the role of interspecific cooperation (Holland et al., 2002). Three main results emerge from our model: (1) there will be complex relationships among environmental quality, intraspecific cooperation, and population dynamics; (2) intraspecific cooperation can facilitate social species to expand their niche width in terms of resource abundance and stability; and (3) social species have greater population resilience to environmental fluctuation than non-social ones. We explain each of these results in greater detail below:

### Complex Relationships Among Environmental Quality, Intraspecific Cooperation, and Population Dynamics

Our results show an unexpected relationship between environmental quality and population size in social organisms. Despite resource scarcity, carrying capacity and the size of a social population can be larger in harsh environments than in benign ones if the benefit of cooperating is high enough. This pattern contradicts the Malthusian view of resource-constrained population dynamics, which does not consider the impact of intraspecific cooperation on population size. It also differs from Boserup’s idea that high population density drives intraspecific cooperation to facilitate population growth, since the degree of intraspecific cooperation is also determined by environmental conditions. In a benign environment, free-riders account for the majority of individuals in a population, and thus there is little benefit of cooperating for further population growth. Therefore, our model synthesizes Malthus’ view of resource-constrained population dynamics (Malthus, 1798) with Boserup’s idea that intraspecific cooperation drives population growth (e.g., *via* agricultural innovation in humans) (Boserup, 1965). We show that environmental quality influences population dynamics both directly (via resource availability) and indirectly (via the degree intraspecific cooperation within the population).

The magnitude of the benefit of cooperation plays an important role in shaping the relationship between environmental quality and population size. When the benefit of intraspecific cooperation is low, the impact of cooperation on population size is weak, and thus the size of a social population—similar to that of non-social one—is mainly determined by environmental conditions. However, when the benefit of cooperation is high, intraspecific cooperation can strongly influence population size. In addition, the degree to which cooperators invest in creating group resources depends on the environmental conditions, such that they contribute more in harsh environments and less in benign environments. Consequently, the direct relationship between environmental quality and population size is less clear than has been previously assumed (Rodenhouse et al., 1997; Rose, 2000).

Two important implications can be drawn from this observation. First, ecologists often assume that better environmental conditions lead to larger populations (Gotelli, 2008; Molles, 2016). Yet, our finding highlights the need to empirically test such assumptions in social species by quantifying lateral forces—the degree of intraspecific cooperation—to understand their impact on population size. Many theoretical and empirical studies have found that social organisms are often more cooperative in harsh environments (Andras et al., 2007; Connelly et al., 2017), yet it remains unknown whether cooperative behavior allows these species to have higher fitness and generate large populations in harsh vs. benign environments. A rare empirical example comes from a study of cooperatively breeding birds, which found that individuals engaged in less social conflict in harsh environments, resulting in higher group productivity (Shen et al., 2012), thus providing some preliminary support for the role of lateral forces on population fitness. Second, the booming human population sizes of the past century are often considered as evidence that human populations are not limited by resources (Steinmann et al., 1998; Kögel and Prskawetz, 2001). However, we caution against such a view (Boserup, 1965), since our model suggests that the effect of resource availability on the degree of cooperation within a population can also influence population dynamics and can constrain population sizes.

Empirically testing the direct and indirect relationships among environmental quality, cooperation, and population dynamics is urgently needed in social species, including our own. Studies examining the Allee effect and how population density of social species influences individual, group, and population fitness have provided some empirical tests of these relationships (Stephens and Sutherland, 1999; Stephens et al., 1999; Angulo et al., 2018). However, the mechanisms (e.g., cooperative and competitive strategies) by which population density affects population dynamics of social species are often unclear (Angulo et al., 2018). Since our model explores the effects of cooperative behavior on population dynamics under different environmental conditions, it can potentially be used to understand the population dynamics of social species that show Allee effects (Angulo et al., 2018), including meerkats (Paniw et al., 2019), wild dogs (Angulo et al., 2013), and Arabian babblers (Keynan and Ridley, 2016). Similarly, the same rule can be applied to interspecific relationships. For example, the stress gradient hypothesis argues that a harsh environment promotes mutualistic relationships between species (Bertness and Callaway, 1994; Callaway et al., 2002). However, how such a mutualistic and competitive relationship over an environmental gradient affects population size is still unclear and deserves further study (Holland et al., 2002; He et al., 2013).

### Intraspecific Cooperation Can Facilitate Social Species to Expand Their Niche Width

If we consider resource availability as a dimension of niche space, we can deduce that social species should have a wider niche breadth than non-social species. If the benefit of cooperating is large, intraspecific cooperation helps social species to maintain positive population growth even when the environments are harsh and have scarce resources. This result is consistent with the social conquest hypothesis (Wilson, 1987, 2012), which argues that ants and humans are the two most dominant species on earth because of their ability to cooperate and form groups (Wilson, 1987, 2012). Despite few formal analyses of this idea, a study in Asian burying beetles showed that social groups are more cooperative in harsher, hotter environments with more intense interspecific competition than in benign, cooler environments where interspecific competitors are absent but intraspecific competition is intense (Sun et al., 2014). As a consequence, social groups are better able than the solitary individuals to expand their thermal niche into harsher environments. Our model supports this idea by predicting that social species should have a wider niche breadth than non-social ones.

### Social Species Have Greater Population Resilience to Environmental Fluctuation

We found that in fluctuating environments, the size of social populations exhibiting intraspecific cooperation is more stable than that of non-social populations that do not cooperate because environment-associated cooperation buffers the impact of environmental fluctuation on social populations. In other words, the key mechanism leading to population resilience of social species is that they are more cooperative in harsh than benign environments. A study of social microbes also found that social populations are more resilient to environmental disturbance (i.e., experimentally lower population density) than non-social ones because more cooperators generate greater group resources (public goods) (Sanchez and Gore, 2013). However, this study assumed that environmental resource availability was stable and the only change in the experiment was lowering population density. Thus, population resilience in this study meant that populations could survive through periods of low density but not environmental harshness *per se*, and that the population dynamics were driven by density-dependent processes such that more cooperators in the population generated greater public goods. Several theoretical studies have investigated this density-dependent process (Epstein, 1998; Hauert et al., 2006; Zhang and Hui, 2011), showing similar patterns of dynamics: abundant public goods generated by cooperators favor the rise of free-riders who do not invest in producing public goods, eventually resulting in an overall reduction of public goods. As public goods become scarce, cooperators are favored by selection again. However, these models exploring the effects of cooperation on population dynamics often assume that environmental conditions are fixed, so that the payoff to cooperators and free-riders is largely determined by the strategies of other individuals. Therefore, such models often find oscillating patterns of evolutionary dynamics of cooperators and free-riders. That is, when there are more cooperators, the free-riders are more likely to encounter the cooperator and get a higher payoff, and thus selection will favor the emergence of free-riders. In reality, however, the benefits gained by the free-riders encountering cooperators should still be influenced by environmental resources. Our results suggest that if reproduction is influenced by resource status, then the number of cooperators and free riders may not show oscillating patterns in the population. Since environmental conditions such as resource availability are assumed constant, these studies cannot determine how bottom-up forces influence the interaction between population and evolutionary dynamics. In contrast, our model suggests that environmental quality can influence both the evolution of intraspecific cooperation and population dynamics, a result that should be incorporated in future empirical studies.

As the earth continues to warm, its climate is becoming increasingly unpredictable (Easterling et al., 2000; Rohr and Raffel, 2010). Some studies have argued that climate change-driven resource scarcity will lead to increased armed conflict in human societies as a result of resource scarcity, a truly neo-Malthusian view (Nordås and Gleditsch, 2007; Scheffran and Battaglini, 2011; Raleigh and Kniveton, 2012). Yet, other studies have argued that the environmental problems caused by climate change will not exacerbate violent conflict (Benjaminsen et al., 2012; Gleditsch, 2012), and may even promote peace and greater cooperation (Slettebak, 2012). Based on our models exploring environmental quality, social interactions, and population dynamics, we predict that harsh environments—those with low resource availability—will also promote more cooperation in human societies depending on the types of cooperative benefits that can be generated under different environmental conditions. Empirical studies testing our model predictions by comparing patterns of intraspecific cooperation under different environmental scenarios, as well as those examining population fluctuation and stability between social and non-social species, will be of great importance for understanding the future dynamics of social species—including our own—in a period of continued global change.

## Data Availability Statement

The datasets presented in this study can be found in online repositories. The names of the repository/repositories and accession number(s) can be found below: https://github.com/YingyuTW/Cooperation_and_Population_Dynamics.

## Author Contributions

Y-YC performed modeling work and analyzed output data. All authors formulated the study, prepared the manuscript, and approved the submitted version.

## Conflict of Interest

The authors declare that the research was conducted in the absence of any commercial or financial relationships that could be construed as a potential conflict of interest.

## Publisher’s Note

All claims expressed in this article are solely those of the authors and do not necessarily represent those of their affiliated organizations, or those of the publisher, the editors and the reviewers. Any product that may be evaluated in this article, or claim that may be made by its manufacturer, is not guaranteed or endorsed by the publisher.
