# Supplementary material for: Cooperation and Lateral Forces: Moving Beyond Bottom-Up and Top-Down Drivers of Animal Population Dynamics
Source: Front Psychol. 2022 Feb 2;13:768773. doi: 10.3389/fpsyg.2022.768773 (PMC8847757; doi:10.3389/fpsyg.2022.768773)
Supplement: Supplementary file 1 [file Data_Sheet_1.pdf]

Supplementary Material

1 Supplementary Table

Table S1. Summary of model parameters.

Name	Value	Description
R_0	[1, 20]	Environmental resource availability, which describes environmental quality.
b_K	[0.1, 6.5]	Cooperation efficiency, representing the benefits of cooperation.
$\emptyset_K$	{0.0, 0.1, 0.2, …, 1.0}	Degree of cooperation, representing an individual's investment in cooperation.
I	40	Maximum resource increment rate: The maximum number of times the resources can be increased by cooperation.
α	3	Maximum reproductive rate of an individual.
β	0.5	The percentage decrease in the reproductive rate caused by per unit cooperative investment
M	1.0	Metabolic consumption of an individual, which represents the amount of energy consumed per unit of time.
K_s	2	Half-saturation constant: The value of the individual energy for reproduction $(s_{i,t} - M)$ at which the reproductive rate $(F_{i,j,t})$ is half of its maximum.
c	0.7	Maximum survival rate.
age_{const}	2	A constant related to age, represents the highest probability of survival of an individual per unit of time.
A	5	The amplitude of environmental fluctuation.
P	1000	The period of environmental fluctuation.
m	0.001	Mutation rate.
N	300	Initial population size.

2 Supplementary Figures

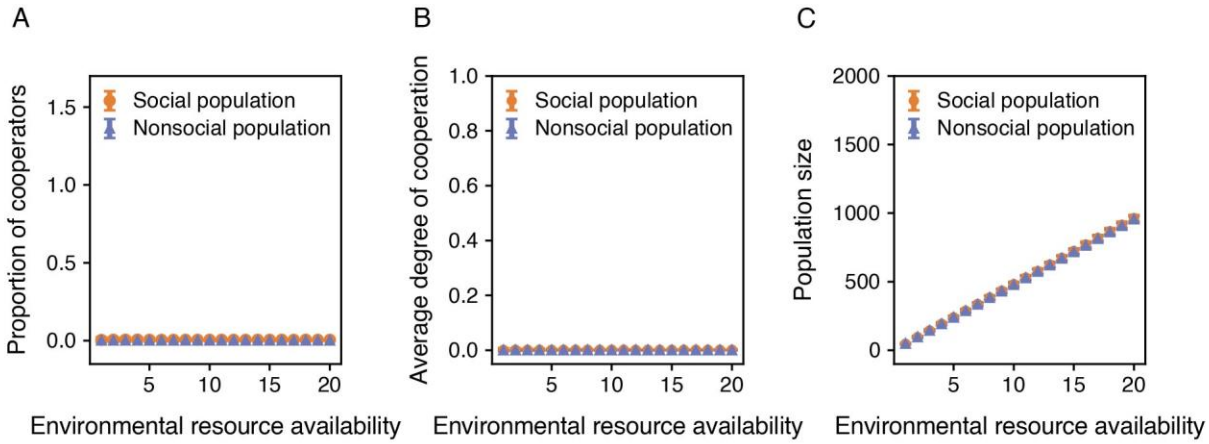

Figure S1. The evolutionary outcome and the population size of non-structured populations. **(A)** The proportion of cooperators, **(B)** the individual average degree of cooperation, and **(C)** the population size in relation to the environmental resource availability in a non-structured population. The proportion of cooperators and the degree of cooperation of non-cooperators are zero.

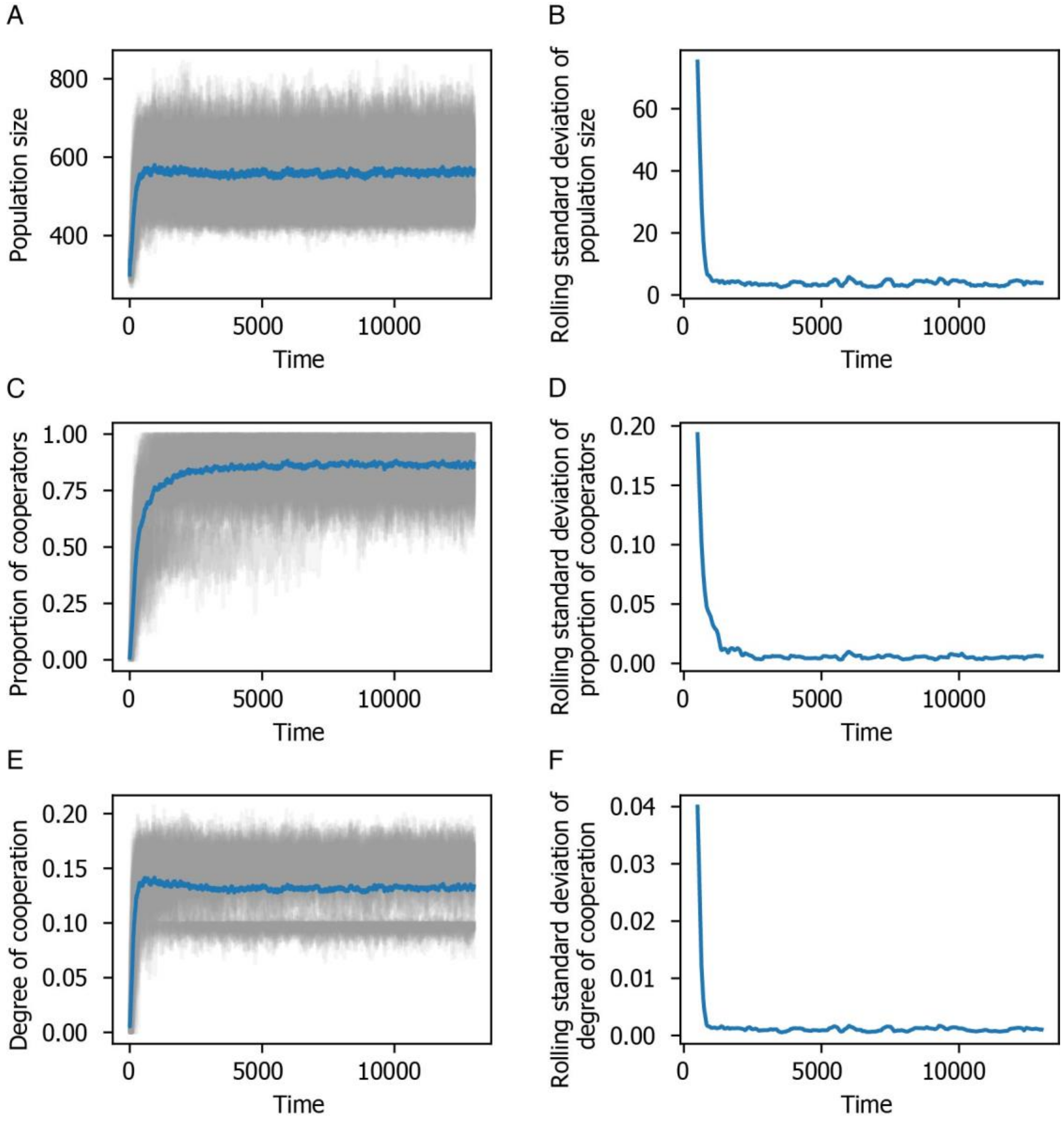

Figure S2. Population and evolutionary dynamics and their rolling standard deviation. Time series of **(A)** population size, **(C)** proportion of cooperators, and **(E)** degree of cooperation and rolling standard deviation of **(B)** population size, **(D)** proportion of cooperators, and **(F)** degree of cooperation of social populations within 13,000 time steps as cooperation efficiency (b_K) is equal to 3 and environmental resource availability (R_0) is equal to 8. Each gray line is derived from one independent simulation, and the blue lines in time series plots are the average of the output data of 100 simulations.

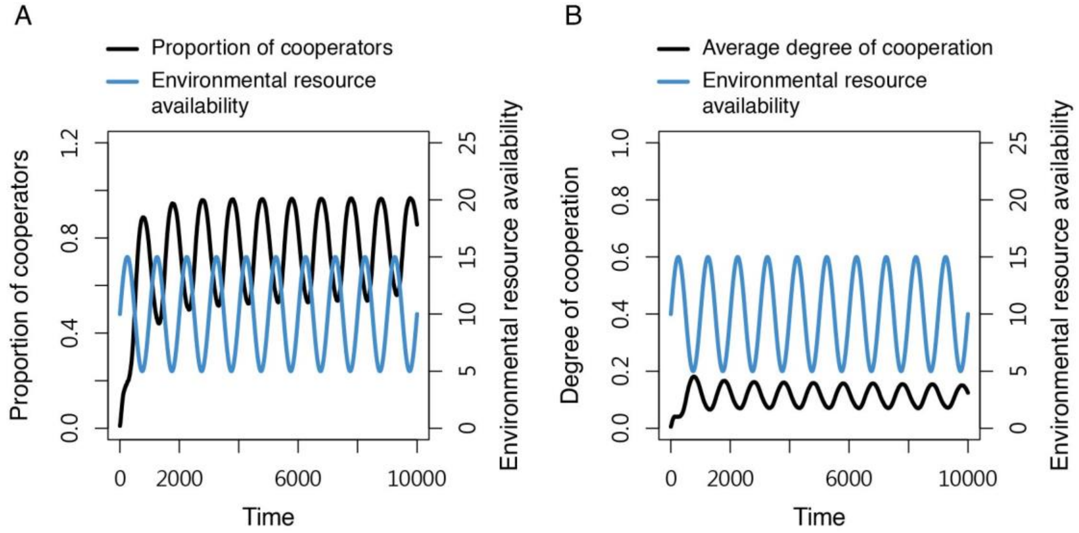

Figure S3. The degree of cooperation changes with fluctuations in environmental resource availability. Time series of **(A)** the proportion of cooperators and **(B)** the individual average degree of cooperation of social populations in a fluctuating environment. Each line is the average of the output data of 500 simulations.
